# Person-centred information to parents in paediatric oncology (the PIFBO study): A study protocol of an ongoing RCT

**DOI:** 10.1186/s12912-015-0120-8

**Published:** 2015-12-21

**Authors:** Anders Ringnér, Maria Björk, Cecilia Olsson, Ulla Hällgren Graneheim

**Affiliations:** Department of Nursing, Umeå University, SE-901 87 Umeå, Sweden; Department of Paediatrics, Umeå University Hospital, SE-901 85 Umeå, Sweden; CHILD Research Group, Department of Nursing Science, School of Health Sciences, Jönköping University, Box 1026, SE-551 11 Jönköping, Sweden; School of Life Sciences, University of Skövde, Box 408, SE-541 28 Skövde, Sweden; Department of Health Sciences, Karlstad University, SE-651 88 Karlstad, Sweden; Department of Health Sciences, University West, SE-461 86 Trollhättan, Sweden

**Keywords:** Childhood cancer, Multi-centre study, Parents, Person-centred information, Process evaluation, Protocol, Randomized controlled trial

## Abstract

**Background:**

Parents of children with cancer experience a demanding situation and often suffer from psychological problems such as stress. Trying to coping with the complex body of information about their child’s disease is one factor that contributes to this stress. The aim of this study is to evaluate an intervention for person-centred information to parents of children with cancer that consists of four sessions with children’s nurses trained in the intervention method.

**Methods/Design:**

This is a multi-centre RCT with two parallel arms and a 1:1 allocation ratio. The primary outcome is illness-related parental stress. Secondary outcomes are post-traumatic stress symptoms, anxiety, depression, satisfaction with information, expected and received knowledge, and experiences with health care providers. A process evaluation is performed to describe experiences and contextual factors. Data are collected using web questionnaires or paper forms according to the parents’ preference, audio recording of the intervention sessions, and qualitative interviews with parents and the intervention nurses.

**Discussion:**

Few studies have evaluated information interventions for parents of children with cancer using large multi-centre RCTs. This intervention is designed to be performed by regular staff children’s nurses, which will facilitate implementation if the intervention proves to be effective.

**Trial registration:**

Clinical trials NCT02332226 (December 11, 2014).

## Background

Parenting a child with cancer is a stressful and demanding situation, and psychological problems such as stress, depression, and anxiety are more common in parents of children with cancer compared to parents of healthy children [1–3]. The difficulties experienced by parents are likely to be not only a consequence of the child’s disease as such, but can also be related to shortcomings in the encounter between these parents and the health care system. This study focuses on information, which should be an integral part of the care but nevertheless is often described as problematic by both parents and health care providers [4–6].

One of the challenges for these parents is to manage the complex and vast body of information available. Also, these parents have ranked their satisfaction with information low in comparison to other aspects of care such as professional skills, availability, and waiting times [7]. At diagnosis, a lot of information is conveyed to the parents, and health care professionals (HCPs) seem to be more attentive to parental needs at this time. However, further into the illness trajectory, parental needs and the HCPs’ focus change and the parents tend to experience less satisfaction with the information they receive [4, 6, 8–10]. Apart from the basic knowledge of the diagnosis and the planned treatment, parents need to look after central venous catheters, deal with the child’s nutrition, and monitor for complications and side effects. Much of this responsibility comes between treatment occasions when the family is out of the hospital [11]. Consequently, interventions for improved information are suggested as a means of protecting parents from excessive stress that in the long run could generate post-traumatic stress symptoms [12].

Although at times questioned, the concepts of family centeredness and child centeredness have had a significant influence in paediatric nursing [13–15]. These concepts imply that either the child or the family as a whole is in focus. However, research has shown that family members also have individual needs of, and preferences for, information [16]. This led us to apply a person-centred approach in this intervention. Using person-centeredness has been shown to improve satisfaction with care due to its individual tailoring of care to the person’s unique needs and to the involvement of the person in their own care [17]. We therefore chose to ground the intervention in this paper on the concept of person-centred information, which we have previously conceptualized as information about social, emotional, existential, and medical topics related to ill health and disease that is grounded in the person’s present knowledge, preferences, and needs and that seeks to empower the person to participate in the care of the sick individual [18].

The evidence base concerning interventions to improve parental wellbeing is not extensive [19, 20]. In general, available interventions tend to be psycho-educational in nature and to teach ways of coping rather than aiming at providing better information about the child’s disease [21–25]. Only a few studies have used interventions aimed specifically at improving and individualizing the information to parents, and the effect on psychosocial distress in those studies has not been consistent [18, 26, 27].

This paper describes an intervention for person-centred information for parents built on *The representational approach to patient education* [28]. Before starting this study, we completed a pilot study. Eight parents participated in the intervention, which was evaluated using a single-case experimental design and qualitative interviews. The pilot study showed that parents were appreciative of the intervention because it gave them an opportunity to discuss their own questions in depth. However, there were no changes in the outcome variables over time, which was likely due to a bias in sampling [18].

### Hypothesis and aims

The aim of this paper is to describe the design of a multi-centre randomized controlled trial that longitudinally evaluates an intervention with person-centred information for parents of children with cancer.

The hypothesis of the study described here is that an intervention with person-centred information emanating from the parents’ own information needs and their current knowledge is associated with reduced illness-related parenting stress, reduced post-traumatic symptoms, reduced depression, reduced anxiety, increased received knowledge, higher satisfaction with information, and reduced number of health care contacts among the participating parents compared to a control group that receives standard care.

A secondary aim of the study described in this paper is to explore the parents’ and intervention nurses’ experiences of participating in the intervention and to describe the contextual factors that influence the intervention’s effect.

## Methods/Design

### Design

The study is a multi-centre randomized controlled trial with two parallel arms and a 1:1 allocation ratio. One arm receives the intervention plus standard care, and the other arm only receives standard care according to local routines at each ward. In parallel to the intervention, a process evaluation is performed to describe experiences and contextual factors of importance, such as intervention dose and fidelity to the protocol.

Both quantitative data (questionnaires – either online or on paper according to the parent’s preference) and qualitative data (interviews, reflective notes, and audio recordings of the intervention meetings) are currently being collected and analysed. The questionnaires are sent out at baseline (T0), once during the intervention (T1), and at two weeks (T2), two months (T3), six months (T4), and one year (T5) after the intervention. A schedule of the intervention is provided in Table 1.

**Table 1:** Weekly schedule of the trial

WEEK	0	4	6	7	8	11	14	20	22	24	30	46	74
Enrolment													
Diagnosis	X												
Eligibility screening		X											
Informed consent		X											
Allocation				X									
Assessments													
Baseline			T0										
Follow-up measurements								T1		T2	T3	T4	T5
Qualitative interviews^a^							Q1			Q2			
Recording of intervention meetings^a^					X	X	X		X				
Intervention													
Intervention meetings					X	X	X		X				

^a^With a subset of participants

### Participants

We are currently recruiting participants from two paediatric oncology tertiary care centres in Sweden. These centres cooperate with local hospitals where some of the treatment and supportive care is given; however, diagnoses and major treatments are performed at the centres. Inclusion criteria are being a parent of a child that a) is diagnosed with a first-time occurrence of a malignancy that is curatively treated and b) was diagnosed within the past two months. Furthermore, parents must be able to speak, read, and write Swedish well enough to participate without an interpreter.

All parents, including any stepparents, are being invited to participate, and interventions and measurements are being performed individually. Eligible parents are approached by a recruitment nurse at each site. After receiving information about the study, informed consent is obtained from parents willing to participate and the baseline questionnaire is distributed.

When the baseline questionnaire has been filled in, parents are randomized to one of the two study arms. Parents of the same child are randomized to the same arm to avoid contamination within couples. The allocation sequences were generated using the online service www.randomization.com. The allocation is performed by an independent person using opaque, pre-numbered envelopes. For each site, two strata are used: i) one participating parent per family and ii) two or more participating parents per family. This strategy will balance the number of parents equally between sites and arms. Both strata are blocked with randomized, varying block sizes (2, 4, or 6 units) that are blinded to the researchers and the person performing the allocation.

### Sample size

According to a power calculation, 130 parents in total will be needed if we expect a moderate effect size (Cohen’s *d* = .5, which corresponds to 13 points of the total score of the primary outcome measure, the Pediatric Inventory for Parents (PIP)), α = .05, and statistical power = .8. We expect that 30 % of parents will not finish the study, meaning that 180 parents in total need to be recruited.

For the qualitative interviews, we plan to interview one third of the parents (*n* = 20) allocated to the intervention arm.

### The intervention

The intervention is based on *the Representational Approach to patient education* developed by Donovan and co-workers. This approach combines two theories. The *common sense model of illness representations* provides a framework for a thorough assessment of the parents’ knowledge of the topic in question. The mental representation of an illness consists of the following six dimensions: identity, cause, timeline, consequences, cure/control, and emotion. Before giving information, the parents’ representations are assessed by the nurse to find gaps, misunderstandings, or confusion in those representations. This then guides the nurse in tailoring the education session for each individual. To alter a representation, another theory about *conceptual change* is used, and this implies that it is crucial to understand the consequences from the knowledge gaps or confusion before providing information to the patient. This method has been used previously in adult services such as oncology and cardiac surgery [28, 29] and was also shown to be useful in the pilot testing of this intervention [18].

Two registered children’s nurses at each site have been employed part-time to deliver the intervention. All have specialist training in paediatric nursing and several years of experience in paediatric oncology. Before the intervention started, they took part in a three-day training workshop that included both theoretical sessions and practical training in the intervention method. They were also provided with a booklet outlining the principle content of the method (available from the authors).

Each parent in the intervention arm has four meetings with the intervention nurse, and before each meeting the parent chooses a topic of interest. A list of suggested topics is available for this, based on topics chosen by parents in the pilot study and during a workshop with clinically experienced nurses in paediatric oncology. The parent can also pick a topic on their own or get suggestions for a topic from the intervention nurse. The meeting, which can be face-to-face or over the telephone according to the parents’ preference, is structured around the Representational Approach, that is, it assesses the representations, explores the gaps, errors, and confusions in those representations, introduces new information, sums up the new information, and sets goals. In parallel, parents receive standard care according to local procedures.

The intervention starts two months after the child has an established diagnosis. This point in time was chosen because previous research has shown that both parents and HCPs report that the most intense information provided at diagnosis has usually ceased being given by this time [4, 6, 30]. The meetings take place at approximately 8, 11, 14, and 22 weeks after the child’s diagnosis.

### Outcomes and measurements

The primary outcome for this study is illness-related parental stress as measured by the PIP [31]. Further, secondary outcomes measured are post-traumatic stress symptoms, anxiety, depression, satisfaction with information, expected and received knowledge, experiences of health care staff, and the number of health care contacts. Background demographic data is also being collected.

The PIP was originally developed in a paediatric oncology setting, but it has been used widely in different paediatric populations. It consists of 42 items that measure both the frequency and difficulty of the following four domains of stress: communication, emotional functioning, medical care, and role functioning. Each item is answered twice on a 5-point Likert-type scale, once fore frequency and once for difficulty. The range for the sum score is 42–210 points, where higher scores indicate higher frequency/difficulty [31]. The PIP has sound psychometric properties and is available in Swedish.

The *Impact of Event Scale-Revised* (IES-R) is used to measure post-traumatic stress symptoms [32]. The dimensions of intrusion, avoidance, and hyperarousal are measured on a total of 22 items with a 5-point Likert-type scale. The sum score ranges from 0 to 88 points, and higher values indicate more stress symptoms. A Swedish translation is available, and the instrument has been used in paediatric oncology previously.

*Anxiety* and *depression* are measured with two visual-digital scales. VDS-Anxiety has been used previously in this population, and it correlates with other instruments such as Spielberg’s State-trait-anxiety inventory. Parent are asked to assess if they have experienced anxiety during the last week on a 7-point Likert-type scale ranging from never to always. VDS-Depression is the equivalent measure for depression [33].

Parental *satisfaction with information* is also measured by visual digital scales. The questions are, for example, “How satisfied are you with the information you have about your child’s illness?” and “How satisfied are you with the information you get from the information sessions?”.

*Expected knowledge and received knowledge* are measured with two instruments, Knowledge Expectations of Significant Others and Received Knowledge of Significant Others [34]. Both instruments measure knowledge in 40 areas. Swedish translations are available, and the instruments have been used in several patient education studies.

*Experiences with your health care provider* are measured on a 15-item instrument developed from Swanson’s theory of caring. This instrument measures aspects of the meeting with the intervention nurse using words such as “comforting”, “informative”, “listening”, and “respectful”. The instrument has previously been translated into Swedish, and psychometric testing of the instrument is in progress.

We also ask the parents for the number of *contacts with the health care* system and what questions were asked by the parents during these contacts. *Summaries of the intervention meetings* are written by the intervention nurses after each meeting. These include the topic that was discussed, the duration of the meeting, what components of the representational approach were used, technical problems, and so on.

Parents also report *background demographic data* such as age, occupation, position in the family, and previous experiences of cancer illness. For the ill child, data about age, sex, and diagnosis are collected.

### Data collection

Data for the instruments are collected using the open-source web survey application LimeSurvey version 2.05. Invitations and reminders are sent out via e-mail and mobile text messages, and the questionnaires can be answered on a computer, tablet, or smart phone by following a hyperlink in the text message or e-mail. Paper questionnaires are provided for participants preferring that.

### Process evaluation

Contemporaneously with the evaluation of the effect of the intervention, we perform a process evaluation. This aims at understand how and why the intervention does or does not work and to describe experiences of parents as well as the intervention nurses [35]. In this part, we will examine how the intervention is performed by the nurses and how well they adhere to the intervention manual via self-reporting and the audio recordings of interview sessions. Approximately one third of the parents participating in the intervention arm are followed more closely during the intervention through recurrent qualitative interviews about their experience of participating. All intervention nurses will participate in focus group interviews. Each intervention meeting is also documented by the intervention nurses, including reflections on the intervention process.

### Data analysis

Quantitative data will be analysed using the SPSS statistical software package. Continuous variables will be reported as the arithmetic mean ± standard deviation, and categorical variables will be reported as the frequency and percentage. Differences between the trial arms will be evaluated using Student’s *t*-test for the outcome variables at the available time points. We will also perform regression analyses using data from the process evaluation, including intervention fidelity and other contextual factors. By using multivariate techniques, we will adjust for any differences found in the baseline characteristics of the participants. When appropriate, Bonferroni adjustments will be made on the overall significance levels. For all of these analyses, effect sizes will be calculated. All analyses will employ the intention to treat principle, and two-tailed *p*-values ≤ .05 will be considered as statistically significant [36].

Qualitative data, such as individual and focus groups interviews and recorded intervention meetings, will be analysed using qualitative content analysis [37]. This method focuses on similarities and differences in data, which is useful for finding possible variances in intervention performance that might explain differences in outcomes.

### Ethical considerations

So far, no specific risks with educational interventions such as this have been described. The participants are asked to report any disadvantages to us, and those will be described in the scientific reporting of the study. Participants have the option to withdraw from the study at any time. We will obtain written informed consent by all participating parents. The study was approved by the Ethical Review Board in Umeå (Dnr 2014-167-31 M, valid for recruitment at Umeå and Lund University Hospitals), and is registered at Clinicaltrials.gov (NCT02332226, December 11, 2014).

## Discussion

The Person-centred Information to Parents in Paediatric Oncology study (PIFBO in its Swedish acronym) uses a randomized controlled approach to evaluate a person-centred intervention for improving the way that information is provided to parents of children with cancer. In general, clinical trials of nursing interventions are sought for in paediatric oncology [38]. To our knowledge, few studies have evaluated information interventions aimed at parents of children with cancer with large multi-centre randomized designs, which makes this study stand out.

Complex interventions such as this one are characterised by multiple components interacting with each other, and the methods in such interventions are often less standardised [39]. Therefore, integrating a process evaluation in a complex intervention trial is recommended [35]. This will enable us to monitor the intervention closely and find out what components in the intervention might be more or less important and what differences contextual factors make. By combining inductive and deductive strategies, as well as mixed methods, we hope to identify otherwise unknown mediators in the intervention and to provide a broader understanding of how it works.

The intervention was designed to be easy to implement, and it can be performed by regular members of the nursing staff after a short period of training; thus, no extra resources would be needed at the wards to implement the intervention. Should the intervention prove to have a beneficial effect on parental wellbeing, it should be easy to implement as a part of standard care. Although it has been difficult to prove the directionality of such associations, increased parental well-being might also have a beneficial effect on the ill child [40].
